# Effect of *Tamarindus indica* leaf powder on plasma concentrations of copper, zinc, and iron in fluorotic cows

**DOI:** 10.14202/vetworld.2016.1121-1124

**Published:** 2016-10-21

**Authors:** Pinaki Samal, R. C. Patra, A. R. Gupta, S. K. Mishra, D. Jena, D. Satapathy

**Affiliations:** 1 Department of Clinical Veterinary Medicine, Ethics and Jurisprudence, College of Veterinary Science and Animal Husbandry, Orissa University of Agriculture and Technology, Bhubaneswar - 751 003, Odisha, India; 2 Department of Animal Nutrition, College of Veterinary Science and Animal Husbandry, Orissa University of Agriculture and Technology, Bhubaneswar - 751 003, Odisha, India; 3 Department of Animal Reproduction, Gynaecology and Obstetrics, College of Veterinary Science and Animal Husbandry, Orissa University of Agriculture and Technology, Bhubaneswar - 751 003, Odisha, India

**Keywords:** cattle, fluorosis, micro-minerals, tamarind leaf

## Abstract

**Aim:**

The main objective of the study was to determine the deleterious effect of fluoride on plasma trace minerals of fluorotic cattle and to evaluate the effect of *Tamarindus indica* leaf powder toward correction of the same.

**Materials and Methods:**

A total of 30 cattle exhibiting chronic sign of fluorosis and 10 healthy cattle from nonfluorotic area were incorporated in this study. Fluorotic cattle were divided into three equal groups consisting of 10 cattle each. Group I from fluoride free area served as healthy control. The Group II received no treatment and served as disease control. Groups III and IV were supplemented with tamarind leaf powder at 15 g and 30 g/day with feed for 60 days. Plasma mineral status was evaluated after 60 days of treatment with double beam atomic absorption spectrophotometer.

**Results:**

Statistical analysis of data revealed a significant (p<0.05) decrease in mean plasma copper (Cu) (0.344±0.007 ppm), zinc (Zn) (0.692±0.06 ppm), and iron (Fe) concentration (1.100±0.01 ppm) in fluorotic cattle in comparison to healthy cattle (0.58±0.010, 2.342±0.04, 1.406±0.04 ppm, respectively). Significant (p<0.05) increase in Cu, Zn, and Fe was recorded after supplementation of tamarind leaf powder to the fluorotic cattle.

**Conclusion:**

It was concluded that fluorotic cattle might be supplemented with *T. indica* leaf powder with feed for the correction of the decreased level of certain plasma minerals.

## Introduction

Fluorotoxicosis/fluorosis is one of the major global health problems affecting both human and animal life and potentiating other health hazards. Excess fluoride (F) intake in mammals exerts toxic effects in many ways including inhibition of enzymes, generation of free radicals and its deposition in various soft tissues as well as hard tissues especially bones and teeth [1]. High levels of fluoride in drinking water have become a potential health hazard in many parts of the world, with approximately 66.62 million victims in India alone [2]. Fluoride bearing rocks are abundant in India from which fluoride leaches out and contaminates the adjacent water source, soil and also the vegetation of that area. Apart from these, the rapid growth of industrialization has added the momentum to the prevalence of disease process. Industrial fluorosis has witnessed a problem of serious concern in many parts of the country. These industrial operations release fluoride in both gaseous and particulate forms. Hydrogen fluoride, silicon tetrafluoride, fluorosilicic acid, and carbon tetrafluoride are the common gaseous forms. Particulate fluoride includes sodium fluoride, fluorspar, cryollite, aluminum-fluoride, and calcium silicate [3]. Thus, industries have been recognized as an important source of development of industrial fluorosis or manmade fluorosis [4].

Fluorine, being the highly electronegative and reactive element, interacts with other minerals in body tissues to produce toxic effects. Earlier studies on laboratory animals revealed alteration in trace mineral status in blood and tissues after fluoride intoxication [5]. Plants have long been used for the treatment of a variety of disorders and for maintenance of good health. Plants and plant products are major sources of therapeutic components that have direct or indirect influence on the physiological systems of the animals. Many plant/herbal preparations have been used to ameliorate the fluoride-induced toxic effects in animals. Protective effect of *Tamarindus indica* L. (tamarind) has been assessed against fluoride toxicity in terms of reducing fluoride concentration in blood and bone and enhancing urinary excretion, in rabbits and rats [6-9] and in blood of cattle under natural fluorosis condition [10]. An anti-hyperglycemic, antihyperlipidemic, antiperoxidative, and antioxidant properties of *T. indica* leaves was evaluated in fluoride intoxicated rats [1].

However, the trace mineral status after supplementation of tamarind to fluorotic cattle has never been assessed. Keeping the above fact into consideration, the present research was aimed to assess the status of copper (Cu), zinc (Zn), and iron (Fe) in fluorotic cattle and its amelioration through *T. indica* leaf powder.

## Materials and Methods

### Ethical approval

The experimental procedures have been conducted in accordance with the guidelines laid down by the Institutional Ethics Committee.

### Study site

This study was conducted in five villages located within 2 km radius of the aluminum smelter plant and 3 km radius of the captive power plant in Talcher-Angul industrial complex of Odisha. This study site is located 133 km away from Bhubaneswar city at latitude 20.83°N and longitude 85.15°.

### Animals and experimental design

About 30 cattle exhibiting chronic sign of fluorosis and 10 healthy cattle from nonfluorotic area were incorporated in this study. Fluorotic cattle were divided into three equal groups consisting of 10 cattle each. Group I from fluoride free area served as healthy control. The Group II received no treatment and served as disease control. Groups III and IV were supplemented with tamarind leaf powder at 15 g and 30 g/day with feed for 60 days.

### Collection of blood

Blood samples from cattle were collected in morning hours by jugular venipuncture. 5 ml of blood was collected and stored in heparinized glass vial (Hi Media, Mumbai) for extraction of plasma. Tubes were marked properly and transported to the laboratory in an ice box.

### Separation of plasma

Plasma samples were separated from the heparinized blood samples after centrifugation at 3000 rpm for 15 min in thermostable refrigerated centrifuge machine (Model 5417R, Eppendorf, Germany) and stored at −40°C for further estimation.

### Plant materials

Tender leaves of *T. indica* were collected from in and around the Bhubaneswar city. The leaves were air dried, grounded to powder with the help of electronic grinder and stored in air tight container. Dried *T. indica* leaf powder (15 and 30 g) was poured into separate zip polythene packet.

### Estimation of micro-minerals

The plasma samples were digested using triple acid (nitric acid, sulfuric acid, and perchloric acid, 4:2:1) mixture and heated below 80°C till digestion. The digested samples were diluted with de-ionized triple glass distilled water and the concentration of Cu, Zn and Fe, was estimated by fully automated double beam atomic absorption spectrophotometer (Model No. AAS4141, Electronics Corporation of India Limited.).

### Proximate principle analysis

Proximate analysis (dry matter, moisture, crude protein crude fiber, ether extract and total ash) of the dried tamarind leaf powder was carried out [11].

### Statistical analysis

Data were analyzed by one-way analysis of variance with *post-hoc* analysis by Duncan’s multiple comparison tests using SPSS 16 software. Results were expressed as mean±SE with p≤0.05 considered statistically significant.

## Results

The dry matter and moisture content of tamarind pulp powder was 90.70% and 9.30%, respectively. The contents of crude protein, ether extract, crude fiber, total ash and total carbohydrate were found to be 14.35%, 4.80%, 12.00%, 7.20% and 56.85%, respectively.

The concentration of Cu, Zn and Fe in plasma of fluorotic cattle of different experimental groups at different observation periods is presented in Table-1. Significant (p<0.05) low level of plasma Cu was recorded in fluorotic cattle (Group II) as compared to healthy cattle (Group I). Non-significant (p<0.05) increase in plasma Cu level was recorded after supplementation of tamarind leaf powder to fluorotic animals (Groups III and IV) from day 30 onward.

**Table-1:** Trace mineral status (ppm) in cattle from fluorotic area along with normal cattle.

Parameters	Days of experiment	Group I healthy cattle	Group II fluorotic cattle	Group III tamarind leaf powder at 15 g	Group IV tamarind leaf powder at 30 g
Cu (ppm)	0	0.580±0.010^B^	0.344±0.007^A^	0.364±0.052^A^	0.348±0.048^A^
	30	0.548±0.007^B^	0.376±0.011^A^	0.402±0.058^A^	0.392±0.053^A^
	60	0.588±0.012^B^	0.368±0.007^A^	0.438±0.058^A^	0.428±0.055^A^
Zn (ppm)	0	2.342±0.04^B^	0.692±0.06^A^	0.746±0.14^A^	0.768±0.13^aA^
	30	2.372±0.06^B^	0.732±0.10^A^	0.894±0.16^A^	0.990±0.16^abA^
	60	2.344±0.03^C^	0.744±0.15^A^	1.028±0.18^AB^	1.200±0.19^bB^
Fe (ppm)	0	1.406±0.04^B^	1.100±0.01^A^	1.108±0.02^aA^	1.104±0.02^aA^
	30	1.302±0.02^C^	1.082±0.01^B^	1.144±0.02^bB^	1.166±0.02^abA^
	60	1.360±0.03^C^	1.102±0.01^A^	1.174±0.02^bB^	1.202±0.02^bB^

Values (mean±SE) having no common superscript (capital letter in a row and small letter in a column) differ significantly at p < 0.05. Zn=Zinc, Fe=Iron, Cu=Copper, SE=Standard error

Fluorotic cattle revealed significantly (p<0.05) low level of plasma Zn as compared to normal cattle (Group I). At the end of the 60 days experiment, non-significant (p<0.05) increase in plasma Zn level was recorded after supplementation of *T. indica* leaf powder at 15 g (Group III), whereas significant (p<0.05) increase in Zn concentration was recorded in plasma of fluorotic cattle supplemented with 30 g of *T. indica* leaf powder.

Fluorotic cattle revealed significantly (p<0.05) low level of plasma Fe level as compared to normal cattle. The plasma Fe level was increased non-significantly (p<0.05) after supplementation of *T. indica* leaf powder at 15 g (Group III) by day 30 and significantly (p<0.05) by day 60 as compared to non-treated fluorotic cattle (Group II). However, supplementation of 30 g *T. indica* leaf powder (Group IV) to fluorotic cattle increased the plasma Fe level significantly (p<0.05) from day 30 onward as compared to cattle received no treatment (Group II).

## Discussion

Significant lower concentration of Cu, Zn and Fe was recorded in cattle reared in the vicinity of aluminum smelter plant.

Decreased levels of serum Cu in this study were similar to the findings reported in sheep [12], goat [13], and bovines [14]. Cu plays a vital role in lipid metabolism in human and animals [15,16]. Plasma concentration of Cu and cholesterol are inversely proposal to each other. Although we did not measure the serum cholesterol level in this study, according to Czerny *et al*. [17] high plasma cholesterol in fluorotic cattle may result decrease plasma Cu level.

Cu and Fe in combination with uric acid play a key role as peroxidation inhibitor and free radical scavenger in antioxidant system of the body [18]. Therefore, a decrease in Cu and Fe in fluorotic cattle may be due to radical removing reactions that are produced by fluorine [13].

A significant decrease in the plasma protein concentration in fluorotic animals may have imparted a lower plasma Zn concentration owing to the importance of amino acids role in the absorption of Zn [19]. The further reasons may be due to the fact that a decreased gastrointestinal absorption along with tissue-specific absorption plays a key role for a lower level of Zn.

Fluoride being highly electronegative halogen has high affinity toward electropositive element. In gastrointestinal tract fluoride form complexes with micro minerals like Cu and Zn and reduce its absorption. Increase in urinary and fecal excretion of various minerals may be another factor responsible for its decrease status in the body.

Supplementation of tamarind leaf powder to fluorotic cattle increased the Fe and Zn concentration. As tamarind not only enhances F elimination but also contains a high amount of Zn and Fe, thus reducing the elimination of these minerals from body [20]. Khandare *et al*. [21] reported the reduced excretion of Zn and Mg by ingestion of tamarind pulp in fluorosis affected human subjects. Intake of tamarind also reported to reduce the level of Zn dependent enzymes like alkaline phosphatase [5].

## Conclusions

Our findings indicate that chronic fluorosis is associated with reduced levels of plasma Cu, Zn, and Fe. Supplementation of *T. indica* leaf powder at 30 g has some effect in plasma Zn and Fe concentration which was observed toward the end of the experiment.

## Authors’ Contributions

This study is a thesis part of M.V.Sc. degree of PS. RCP, ARG and SKM planned the study and PS done the research under the guidance of RCP and ARG. DJ and DS helped in data collection and estimation process of this experimentation. All authors participated in draft and revision of the manuscript. All authors read and approved the final manuscript.
